# Use of Bayesian networks in Brazil high school educational database: analysis of the impact of COVID-19 on ENEM in Pará between 2019 and 2022

**DOI:** 10.3389/fdata.2025.1485493

**Published:** 2025-03-12

**Authors:** Sandio Maciel Dos Santos, Marcelino Silva da Silva, Fábio Manoel França Lobato, Carlos Renato Lisboa Francês

**Affiliations:** ^1^Graduate Program in Electrical Engineering, Federal University of Pará, Belém, Brazil; ^2^Graduate Program in Electrical Engineering, Institute of Engineering and Geosciences, Federal University of Western Pará, Santarém, Pará, Brazil; ^3^Institute of Engineering and Geosciences, Federal University of Western Pará, Santarém, Pará, Brazil; ^4^Graduate Program in Electrical Engineering, Institute of Technology, Federal University of Pará, Belém, Brazil

**Keywords:** COVID-19, ENEM, educational inequality, remote learning, regional disparities

## Abstract

This study examines the impact of the COVID-19 pandemic on academic performance and student participation in the National High School Exam (ENEM) in the state of Pará, Brazil, focusing on the interaction between socioeconomic factors, access to technology, and regional disparities. The research employed a mixed-methods approach, analyzing quantitative data from ENEM results (2020–2022) and qualitative interviews with educators and students. The findings indicate that the pandemic exacerbated pre-existing educational inequalities, particularly affecting low-income students and those enrolled in public schools. The highest dropout rates were recorded among students with a family income of up to one minimum wage, highlighting the barriers posed by limited access to technology and infrastructure for remote learning. A statistical analysis revealed a 20% increase in scores among students with access to computers and the Internet, particularly in private schools. The study also found significant regional differences across Pará's mesoregions, with Marajó and Southeast Pará facing more persistent challenges in reducing dropout rates compared to the Metropolitan Region of Belém. These results underscore the urgent need for region-specific public policies that address disparities in educational resources, including targeted investments in digital infrastructure and teacher training for remote education. The study concludes that comprehensive support programs, including psychological assistance for students, are essential for building a more resilient and equitable educational system capable of withstanding future crises.

## Introduction

The COVID-19 pandemic brought transformative changes across various sectors, including healthcare, education, and urban infrastructure. Its impact exposed and exacerbated preexisting social inequalities, shaping how different groups navigated the challenges posed by the crisis. In education, the shift from in-person to remote learning introduced significant difficulties, including limited access to technology and the internet, increased stress levels, and disruptions to the learning environment—challenges that were particularly pronounced among low-income students in developing countries such as Brazil.

The pandemic most affected the education sector, leading to an abrupt transition from in-person to remote learning. Students worldwide face significant challenges, including limited access to technology and the internet, difficulties concentrating, and increased stress due to changes in the learning environment (Alqahtani and Rajkhan, 2020; Zhu et al., 2022). In countries like Brazil, these challenges were exacerbated by socioeconomic inequalities that directly impacted how students accessed and benefited from remote learning (Ferreira et al., 2022; Silva and Ribeiro-Alves, 2021).

Recent studies have demonstrated that the shift to remote learning, particularly in developing countries, led to significant learning losses, disproportionately affecting low-income students who lacked adequate technological resources to engage in virtual classes (Van Lancker and Parolin, 2020). Other studies have emphasized the crucial role of educational policies implemented during the pandemic in mitigating these impacts, highlighting that interventions providing access to technology and psychological support are essential for reducing educational inequalities (Bartholo et al., 2023).

This study aims to examine the impact of the COVID-19 pandemic on the academic performance of high school students in Pará, focusing on an analysis of microdata from the National High School Exam (ENEM) from 2019 to 2022. While the pandemic introduced new challenges, including prolonged school closures and remote learning, this study seeks to identify the key social factors that influenced student performance during this critical period.

The analysis examines the correlation between factors such as household income, parental educational level, and access to technological resources with academic performance, aiming to understand how these elements contributed to variations in ENEM scores before and during the pandemic.

References to infection rates are maintained in this study to illustrate how infection peaks and social restriction measures, such as lockdowns, affected the learning environment, particularly in regions with high levels of social inequality. Previous studies indicate that these restrictions disproportionately impacted students from low-income families, who had limited access to educational resources (Hawkins et al., 2020; Park and Awan, 2023). Therefore, understanding the relationship between infection rates and the educational policies implemented during these periods is crucial for contextualizing the challenges students faced throughout the pandemic.

Studies conducted in other countries, such as Nigeria and China, have shown that social factors, including family composition and income, directly influence academic performance in remote learning contexts (Ariyo et al., 2022; Zhu et al., 2022). In Brazil, the pandemic highlighted regional and socioeconomic inequalities, which were reflected in students' performance on national exams such as the ENEM (Weber Neto et al., 2022; Gonçalves and Pereira, 2024).

The study by Livingston et al. (2022) reveals that the COVID-19 pandemic exposed inequalities in digital access to education, with the lack of adequate infrastructure hindering remote learning in various regions. The research emphasizes the urgent need for investments in digital inclusion to address these disparities, a challenge that is equally relevant for Brazil and its diverse regions. This study contributes to the literature by examining how these factors specifically manifested in Pará, a region with unique socioeconomic characteristics within the Amazonian context.

## Methods

The methodology employed in this study involves the application of data science techniques, specifically Educational Data Mining (Filatro, 2021; Mouromtsev and d'Aquin, 2016), as the primary approach for knowledge extraction from databases, utilizing the gathered information to support decision-making processes. The analysis focuses on educational data from high school students and graduates to investigate the impacts of the COVID-19 pandemic.

For this study, datasets from the ENEM exams for the years 2019 (pre-pandemic period) and 2020–2022 (pandemic period) were selected. These years were chosen due to the significant increase in COVID-19 infections, alongside the corresponding school censuses for the same periods, which serve as sources of microdata for ENEM. This selection allows for an examination of student performance amid the challenges posed by the pandemic, particularly in the context of national exam responses, with the aim of determining the influence of school closures during periods of high epidemic risk (Pereira Junior et al., 2021; Karakose, 2021; Reimers, 2022).

The ENEM microdata for 2019 and 2022 consists of datasets of 2.24, 1.88, and 1.40 gigabytes, respectively, each containing a set of 76 variables. Together, these datasets represent over 14 million instances, corresponding to the number of exam participants nationwide. Among the 76 analyzed variables, 22 were selected based on their stronger correlation with performance scores, as presented in Table 1. This selection was made to optimize the construction of the representative Bayesian Network (BN) for the problem at hand (Murphy and Russell, 2002).

**Table 1:** Socioeconomic and academic variables in educational data analysis.

**Parameter**	**Variables**	**Description**
Sex	TP_SEXO	Sex
Color and race	TP_COR_RACA	Color and race
Dependence_ADM	TP_DEPENDENCIA_ADM_ESC	Administrative dependency
Abstention	TP_PRESENCA_CN	AE natural sciences
Abstention	TP_PRESENCA_CH	AE human sciences
Abstention	TP_PRESENCA_CL	AE code and language
Abstention	TP_PRESENCA_MT	AE mathematics and technology
Performance	NU_NOTA_CN	Score in natural sciences
Performance	NU_NOTA_CH	Score in human sciences
Performance	NU_NOTA_CL	Score in code and language
Performance	NU_NOTA_MT	Score in math and technology
Father's education	Q001	Father's level of education
Mother's education	Q002	Mother's level of education
Father's occupation	Q003	Father's occupation
Mother's occupation	Q004	Mother's occupation
People household	Q005	${\text{N}}^{\underline{o}}$. of people in the household
Family income	Q006	Average family income
Bathrooms	Q008	${\text{N}}^{\underline{o}}$ of bathrooms
Bedrooms	Q009	${\text{N}}^{\underline{o}}$ of bedrooms
Computer	Q024	Computer
Internet	Q025	Internet

**AE**, attendance in the exam, ${\text{N}}^{\underline{o}}$, **number**.

Unlike previous studies that relied solely on average scores as a performance criterion (Boneti and de Oliveira, 2017; Ferrari Bravin et al., 2019; Vinicios do Carmo et al., 2021; da Silveira et al., 2015), this study adopts a more comprehensive approach. Bayesian Networks were selected for their ability to model complex probabilistic relationships and incorporate latent variables that may influence student performance. While traditional metrics, such as Pearson or Spearman correlations, are useful for measuring linear and monotonic associations between variables, Bayesian Networks provide a more flexible approach for identifying non-linear dependencies and causal inferences, facilitating a more detailed analysis of interactions between sociodemographic variables and academic performance.

### Data preprocessing

The data were cleaned to remove inconsistencies and fill in missing values. Categorical variables were encoded, and continuous variables were normalized to facilitate analysis.

### Performance stratification

The study categorizes performance using quartiles, calculated based on the minimum and maximum score values in each knowledge area (Bendikson et al., 2011; Waheed et al., 2019), while also considering the number of dropouts per exam edition ξ. As shown in Equation 1:


(1) $$K_{Q}\;\frac{P(\vec{u}+1)}{4}$$

To calculate the position of the *K*_*Q*−th_ quartile in an ordered dataset, where:

- *P* represents the percentile (in the case of quartiles, *P* ranges from 1 to 3, corresponding to the first, second, and third quartiles).
- $\vec{u}$ is the total number of observations.

Table 2 illustrates the discretization into three groups using the quartile method. The K_Q ≤ 25%_ group represents students with performance below 25%, _25%_ < K_Q < 75%_ includes those with scores between 26 and 74%, and K_Q ≥ 75%_ encompasses students with performance above 75%. The variable ξ refers to the number of dropouts per exam edition. This categorization is essential for understanding the real impacts of COVID-19 on sociodemographic dimensions and its influence on student performance during educational disruptions.

**Table 2:** Distribution of ENEM participants by socioeconomic parameters and dropout rate (2019–2022).

**Edition**	**ξ**	**K_*Q* ≤ 25*%*_**	**_25*%*_ < K_*Q*__ < 75*%*_**	**K_*Q* ≥ 75*%*_**
2019	Inf%	–inf−443	444–546	557–inf+
2020	Inf%	–inf−439	440–544	545–inf+
2021	Inf%	–inf−443	444–572	573–inf+
2022	Inf%	–inf−484	484–602	603–inf+

inf%, absent participants; –inf, scores below 25%; inf+, scores above 75%.

### Modeling with Bayesian networks

Bayesian Networks were constructed using the PGMPY library (Ankan and Panda, 2015), chosen for its ease of configuration and usability, as well as its intuitive generation of probabilistic relationships and display of Conditional Probability Tables (CPTs) for each node. Visualization was facilitated by the pyAgrum API (Ducamp et al., 2020).

### Selected variables

The variables representing scores in different knowledge areas were grouped into four performance analysis groups, as described in Table 2. For the Monthly Household Income variable (Q006), which consists of income ranges (e.g., “from R$0.00 to R$998.00”), the lowest salary and the number of people per household (Q005) were used to replace the original text and group them according to the ENEM variable dictionary (Brasil, 2022).

The data used in this study were obtained from the public ENEM microdata and are available for consultation through the microdata^1^ repository. This allows other researchers to replicate the analysis, promoting transparency and validation of results.

While Bayesian Networks offer a significant advantage in capturing complex relationships, they have inherent limitations, such as the requirement for conditional independence assumptions between variables when employing the Hill-Climb Search algorithm (Koller and Friedman, 2009). To mitigate these limitations, a structure validation analysis was performed using scoring metrics such as K2Score, BicScore, and BdeuScore to ensure the robustness and reliability of the results, as shown in Table 3 (Koller and Friedman, 2009). These metrics provide quantitative measures of the network's structural quality, balancing model fit and complexity.

- **K2Score**: Higher values indicate a better fit under the K2 metric, reflecting how well the structure aligns with the data.
- **BicScore** and **BdeuScore**: Negative values reflect penalization for model complexity, which helps prevent overfitting by discouraging overly complex structures that do not significantly improve the model's performance.

**Table 3:** Score comparison for Bayesian network structures.

**Edition**	**K2Score**	**BicScore**	**BdeuScore**
2019	1.32 × 10^7^	4.99 × 10^5^	−4.98 × 10^5^
2020	7.34 × 10^6^	−3.68 × 10^5^	−3.66 × 10^5^
2021	1.06 × 10^7^	−3.14 × 10^5^	−3.11 × 10^5^
2022	1.54 × 10^7^	−3.95 × 10^5^	−3.94 × 10^5^

Table 3 compares the scores for different Bayesian Network structures across multiple editions, providing a quantitative basis for evaluating model robustness. Higher K2Score values indicate a better fit, while BicScore and BdeuScore values reflect the trade-offs between accuracy and simplicity. These metrics are instrumental in validating the network structure, ensuring that it captures underlying dependencies without overfitting or introducing unnecessary complexity.

However, the absence of a detailed discussion or interpretation of these scores limits the understanding of their implications for structure validation in Bayesian Networks. Future research should build on these findings by incorporating a comprehensive analysis of the scoring metrics and exploring their theoretical and practical impacts. Additionally, qualitative analyses or empirical validations should complement these results, offering further insights into the model's performance and applicability in real-world scenarios.

The statistical and probabilistic inferences drawn from the ENEM microdata and the School Census aim to compare the sociodemographic effects of successive epidemic outbreaks, confirmed cases, and deaths on student performance. This comprehensive approach seeks to identify those most likely to be affected when a public health alert is declared.

## Results

The findings of this study reveal significant trends regarding the impact of the COVID-19 pandemic on academic performance and student participation in the Brazilian National High School Exam (ENEM) in the state of Pará, Brazil, from 2019 to 2022.

As shown in Table 4, participants with a household income below the minimum wage exhibited the highest dropout rates from ENEM in 2020 and 2021 compared to 2019. These data underscore the disproportionate impact of epidemic outbreaks, such as the COVID-19 pandemic, on low-income populations, where prolonged public institution closures directly hindered educational access for these groups (Dutra et al., 2023; Ferreira et al., 2022; Torres et al., 2020). This impact reflects a scenario where socioeconomic conditions restrict access to remote learning alternatives, particularly in more vulnerable regions.

**Table 4:** Dropout percentage of ENEM participants by socioeconomic parameter (2019–2022).

**Parameters**	**ENEM edition**							
	**2019**		**2020**		**2021**		**2022**	
ADM dependence	Public	15.45	Public	41.49	Public	26.78	Public	33.52
Color and race	Brown	69.16	Brown	66.84	Brown	64.25	Brown	64.71
Mother's education level	Elementary	39,85	Elementary	35.51	Elementary	30.60	Elementary	32.72
Father's education level	Elementary	45.44	Elementary	42.37	Elementary	38.53	Elementary	24.43
Mother's occupation	Group 2	46.07	Group 2	46.98	Group 2	47.11	Group 2	46.07
Father's occupation	Group 1	37.24	Group 1	32.31	Group 1	28.77	Group 1	31.21
Number of people	+4	45.10	+4	41.31	+4	41.07	+4	49.45
^*^Family income	[0-1] Salary	63.73	[0-1] Salary	70.00	[0-1] Salary	62.41	[0-1] Salary	67.36
Number of bathrooms	1	87.35	1	83.74	1	82.15	1	81.51
Number of rooms	2	51.39	2	51.89	1	82.15	2	51.02
Computer	No	87.80	No	84.10	No	80.88	No	83.90
^*^Internet	No	64.79	Yes	52.46	Yes	69.42	Yes	70.31

ADM, Administrative; Elementary, Elementary School; High, High School; and Group, Occupation details in the table.

The data presented in Table 4 reveal a concerning trend of increasing dropout rates among low-income participants during the years most impacted by the pandemic. This observation suggests that socioeconomic inequalities were exacerbated during this period, particularly for individuals reliant on public institutions who faced greater challenges in adapting to remote learning.

Further analysis of participants scoring above 75% shows that students attending or who had attended private schools during the pandemic performed better than their public school counterparts. These findings suggest that resource availability, such as access to computers and the internet, played a crucial role in academic success, especially during remote learning periods. Table 5 highlights a clear relationship between access to these resources and higher exam scores. For instance, private school participants with internet access exhibited an average performance increase of 20% compared to their peers in public schools.

**Table 5:** Academic performance of participants scoring above 75% in the ENEM by socioeconomic parameter (2019–2022).

**Parameters**	**ENEM edition**							
	**2019**		**2020**		**2021**		**2022**	
ADM dependence	Private	66.36	Private	52.05	Private	53.70	Private	43.05
Color and race	Brown	59.55	Brown	54.76	White	50.69	Whine	47.68
^*^Mother's education level	High	37.26	High	37.93	High	35.98	High	37.50
Father's education level	High	43.39	High	40.58	High	38.03	High	35.04
^*^Mother's occupation	Group 4	28.11	Group 4	37.91	Group 4	38.58	Group 4	48.47
Father's occupation	Group 4	38.79	Group 4	41.22	Group 4	43.61	Group 4	42.16
Number of people	4	35.58	4	39.40	4	40.20	4	39.85
Family income	[0-1] Salary	32.23	[0-1] Salary	20.20	[0-1] Salary	15.20	[0-1] Salary	19.64
Number of bathrooms	1	61.56	1	48.60	1	44.24	1	37.15
Number of rooms	2	48.85	2	44.84	2	43.67	2	43.53
^*^Computer	Yes	47.91	Yes	65.83	Yes	64.42	Yes	80.64
^*^Internet	No	70.78	Yes	87.04	Yes	71.64	Yes	97.91

ADM, Administrative; Elementary, Elementary School; High, High School; and Group, Occupation details in the table.

The data suggest that access to technological resources significantly impacted academic performance during the pandemic. Students with home access to a computer and internet achieved higher scores, underscoring the importance of ensuring adequate infrastructure for remote learning, particularly during periods of school disruption.

The data also indicate that higher maternal employment and education levels correlated with improved student performance. This finding suggests that the home environment can substantially influence academic outcomes beyond direct access to material resources. Parental involvement and education provide additional support, either by fostering a more structured study environment or by promoting the value of continuous learning (Fernandes et al., 2023; Navarro et al., 2021).

**Figure 1:**
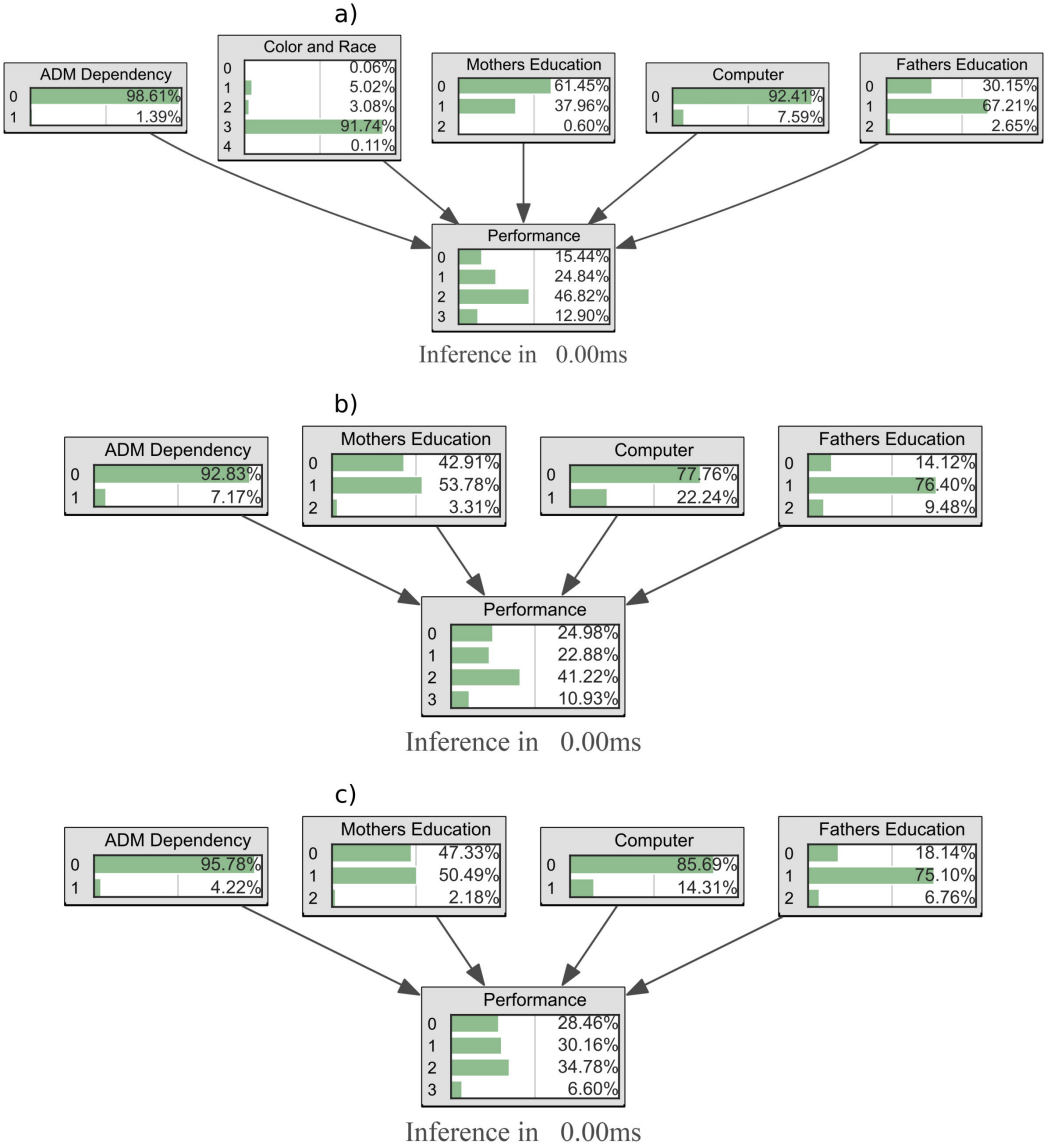
Bayesian network with ENEM Data from 2019 to 2022: Pre- and Post-COVID-19 analysis. **(A)** Bayesian inference for in 2019. **(B)** Bayesian inference for in 2021. **(C)** Bayesian inference for in 2022.

This initial analysis aimed to clarify the influence of social parameters on the ENEM performance of participants in Pará. A Bayesian probabilistic analysis was conducted to investigate how the rise in respiratory syndrome cases during the COVID-19 pandemic affected student performance. This analysis employed techniques such as Hill-Climb Search, K2 Score, and Variable Elimination, supported by the pyAgrum library, to visualize Bayesian Networks from 2019 to 2022, as illustrated in Figure 1.

The Bayesian networks derived from 2019 data underscore key variables significantly impacting ENEM participants' performance, including parental education level, family income, computer access, and the administrative status of the household, as illustrated in Figure 1A. This organizational structure defines a probabilistic dependency flow among selected parameters, establishing a solid foundation for performance analysis.

Applying the same methodology to structure Bayesian networks with educational data from 2021 and 2022 (Figures 1B, C) reveals a marked shift directly influenced by the COVID-19 pandemic: household computer presence no longer emerges as a primary variable of importance. This phenomenon is particularly relevant, considering that the 2020 ENEM occurred amidst substantial educational disruptions, with many students facing challenges in accessing the technology required for remote learning (Guia do Estudante, 2021; de Albuquerque, 2020).

The analysis of 2020 data, therefore, faces unique challenges, as the pandemic unpredictably altered relationships among variables traditionally associated with academic performance. In a context of emergency remote learning and unequal access to resources, the data reflect atypical patterns, with socioeconomic variables such as family income and parental education, becoming even more unstable and less predictable.

Bayesian Networks (BNs) effectively model these complex interdependencies among educational and sociodemographic variables, allowing for causal inferences and identification of latent variables affecting student performance (Murphy and Russell, 2002). However, when dealing with 2020 ENEM data, BNs encounter limitations, as the pandemic's profound impact on low-income students led to record absenteeism and disparities in performance across different socioeconomic contexts (de Andrade and Bocardi, 2024; de Albuquerque, 2020).

This pandemic context highlights the need for critical evaluation of probabilistic models such as BNs. While robust, these networks depend on assumptions of conditional independence that may be compromised under extreme conditions, as imposed by the pandemic. Result interpretation thus requires caution, taking into account the limitations and potential biases within the data (Murphy and Russell, 2002).

Variable selection by the Bayesian network, which identifies the most relevant conditional dependencies, shows that higher levels of parental education correlate with better participant performance, as shown in Table 6 (Biener et al., 2019). However, ENEM dropout rates (ξ) increased by 19% from pre- to post-pandemic periods for parents with only primary education and by 8% for those with higher education. During the 2020 pandemic, dropout rates were ~31% for parents with primary education and 10% for those with higher education. By 2021, these rates decreased to around 14 and 9%, respectively, reflecting a slight recovery in educational conditions.

**Table 6:** Relationship between father's education level and students' academic performance.

**ENEM edition**	**Father's level of education**			
	**Group**	**Elementary school**	**High school**	**University education**
2019	ξ	^*^20.68	13.58	^*^10.88
	*K*_Q ≤ 25%_	30.55	23.05	19.25
	*_25%_ < K_*Q*__ < 75%_*	41.95	49.55	47.39
	*K*_Q ≤ 75%_	6.81	13.82	22.48^*^
2020	ξ	^*^51.36	38.01	29.60^*^
	*K*_Q ≤ 25%_	21.83	19.01	13.24
	*_25%_ < K_*Q*__ < 75%_*	24.05	35.35	36.36
	*K*_Q ≥ 75%_	2.76	7.64	20.80^*^
2021	ξ	^*^34.73	25.30	^*^17.75
	*K*_Q ≤ 25%_	29.34	24.08	19.20
	*_25%_ < K_*Q*__ < 75%_*	32.11	41.82	40.86
	*K*_Q ≥ 75%_	3.82	8.80	20.19^*^
2022	ξ	^*^39.76	27.90	^*^18.92
	*K*_Q ≤ 25%_	35.51	32.06	25.72
	*_25%_ < K_*Q*__ < 75%_*	23.20	34.92	39.88
	*K*_Q ≥ 75%_	1.54	5.12	15.98^*^

Beyond the general analyses, the study also explored regional variations within Pará, as illustrated in **Figure 3**. The Metropolitan Region of Belém and Northeast Pará managed to reduce dropout rates during the pandemic between 2020 and 2021, in contrast to other regions that maintained high dropout rates. This finding suggests possible differences in implementing remote educational support strategies and local infrastructure.

An important aspect to highlight is the conditional probability between administrative dependency and the availability of a computer in the household for educational purposes. The inferences reveal a significant correlation, especially among public school students with computer access, showing a strong association with their ENEM scores. Analyzing the scores of students classified in the K_Q_ < 75% group, there is a marked disparity between those with and without computer access, indicating a significant increase in performance for the former. Specifically, there was a 13% increase among private school students, as shown in Table 7.

**Table 7:** Relationship between administrative dependency and families with computer access at home in the 2019 ENEM.

**Group**	**Administrative dependency**		
	**Public**	**Private**	**Computer**
ξ	16.09	10.79	None
*K*_Q ≤ 25%_	25.92	17.26	None
*_25%_ < K_*Q*__ < 75%_*	47.30	48.25	None
*K*_Q ≥ 75%_	^*^10.68	23.70	None
ξ	7.21	3.51	At least one
*K*_Q ≤ 25%_	11.34	5.29	At least one
*_25%_ < K_*Q*__ < 75%_*	34.57	24.25	At least one
*K*_Q ≥ 75%_	^*^46.87	66.95	At least one

Another crucial aspect to consider is the conditional probability between administrative dependence and the availability of a home computer for educational activities. Inferences indicate a significant correlation, particularly among public school students with computer access, showing notable improvements in ENEM scores compared to those without access. Among students in the _25% < _ K_Q < 75%_ group, a considerable increase in scores is observed for those with computer access. Specifically, private school students showed a 20% increase, as detailed in Table 8.

**Table 8:** Relationship between family income and student performance.

**ENEM edition**	**Family income**							
	**2019**		**2020**		**2021**		**2022**	
**Group**	**C1**	**C6**	**C1**	**C6**	**C1**	**C6**	**C1**	**C6**
ξ	16.22	6.39	42.32	22.53	27.84	12.85	30.96	15.79
*K*_Q ≤ 25%_	22.51	10.41	20.35	8.07	26.49	9.50	33.96	14.71
*_25%_ < K_*Q*__ < 75%_*	47.01	41.06	31.94	41.59	39.59	39.85	31.61	39.59
*K*_Q ≥ 75%_	(+) 10.25	41.14 (^*^)	(+) 5.39	37.81 (^*^)	(+) 6.08	37.80 (^*^)	(+) 3.47	29.90 (^*^)

C1, Income from zero up to 1 Minimum Wage; C6, more than 3 Minimum Wages.

Moreover, the analysis of family income reported by participants reveals a strong relationship between higher income levels (C6; ^*^) and student scores, as illustrated in Table 8. Consistent with this inference, examining the pre-established family income brackets shows a decline in performance among students reporting incomes up to one minimum wage (C1). Among those scoring in the *K*_*Q* ≥ 75%_ group, there was a notable reduction of ~6.5% in the participants within this income bracket.

**Figure 2:**
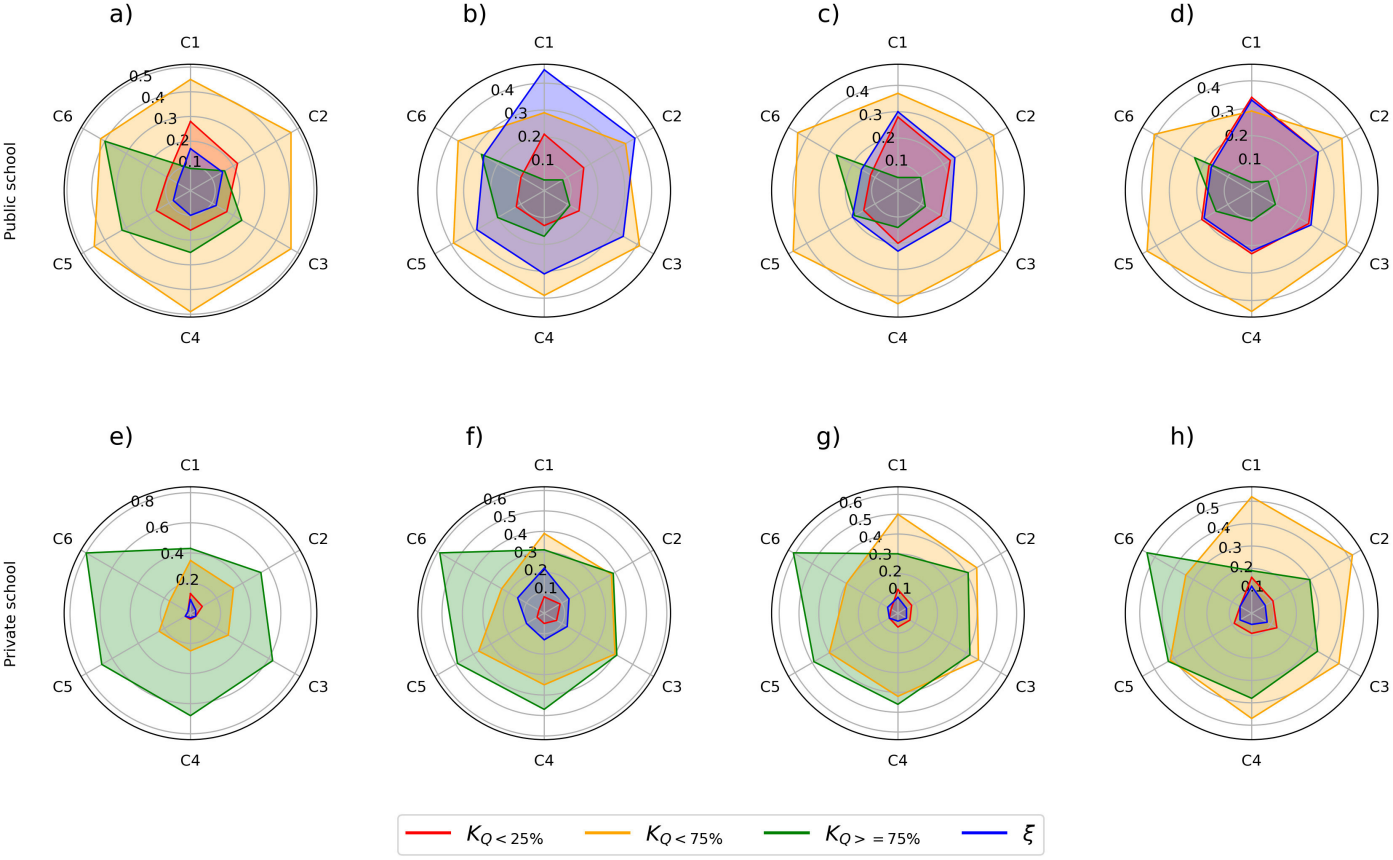
Performance radar of students through family income and administrative dependency. C1: Up to 1 minimum wage; C2: 1.5 minimum wages; C3: 2 minimum wages; C4: 2.5 minimum wages; C5: 3 minimum wages; C6: More than 3 minimum wages. Colors represent performance percentages: Blue: Dropouts; Orange: Scores between [0–25]; Green: Scores between [26–74]; Red: Scores between [75–100]. **(A)** Edition 2019. **(B)** Edition 2020. **(C)** Edition 2021. **(D)** Edition 2022. **(E)** Edition 2019. **(F)** Edition 2020. **(G)** Edition 2021. **(H)** Edition 2022.

A more detailed analysis assessed the impact on performance by considering participants' administrative dependence and family income. It was observed that the proportion of public school students in the K_Q ≥ 75%_ group decreased when associated with incomes up to one minimum wage. Conversely, the dropout rate increased by 30%. Figure 2 provides a visual representation of participant performance based on family income.

Figure 2 shows a notable increase in the number of participants from private schools in the _25% < _ K_Q < 75%_ group between 2020 and 2021. This shift may be attributed to the challenges posed by remote learning during peak COVID-19 case numbers in Brazil. In contrast, most dropouts in the national exam occurred among public school students (Navarro et al., 2021).

**Figure 3:**
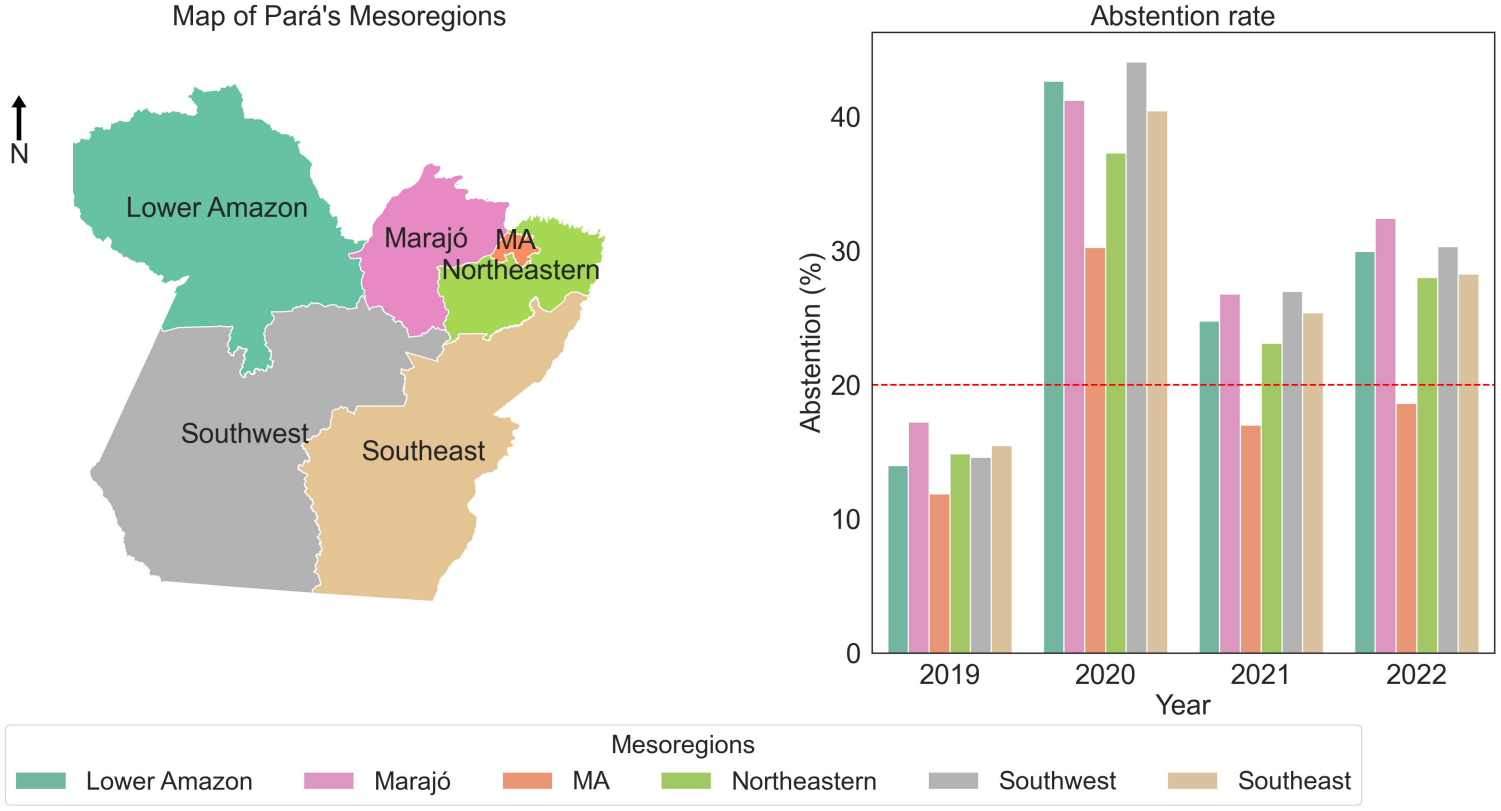
Abstention of students from the state of Pará by mesoregion.

A more specific analysis of educational data from the state of Pará, focusing on the relationship between its six mesoregions and the school census, clarifies whether the impact of the COVID-19 pandemic had uniform effects on dropout rates and the overall performance of participants, as shown in Figure 3.

**Figure 4:**
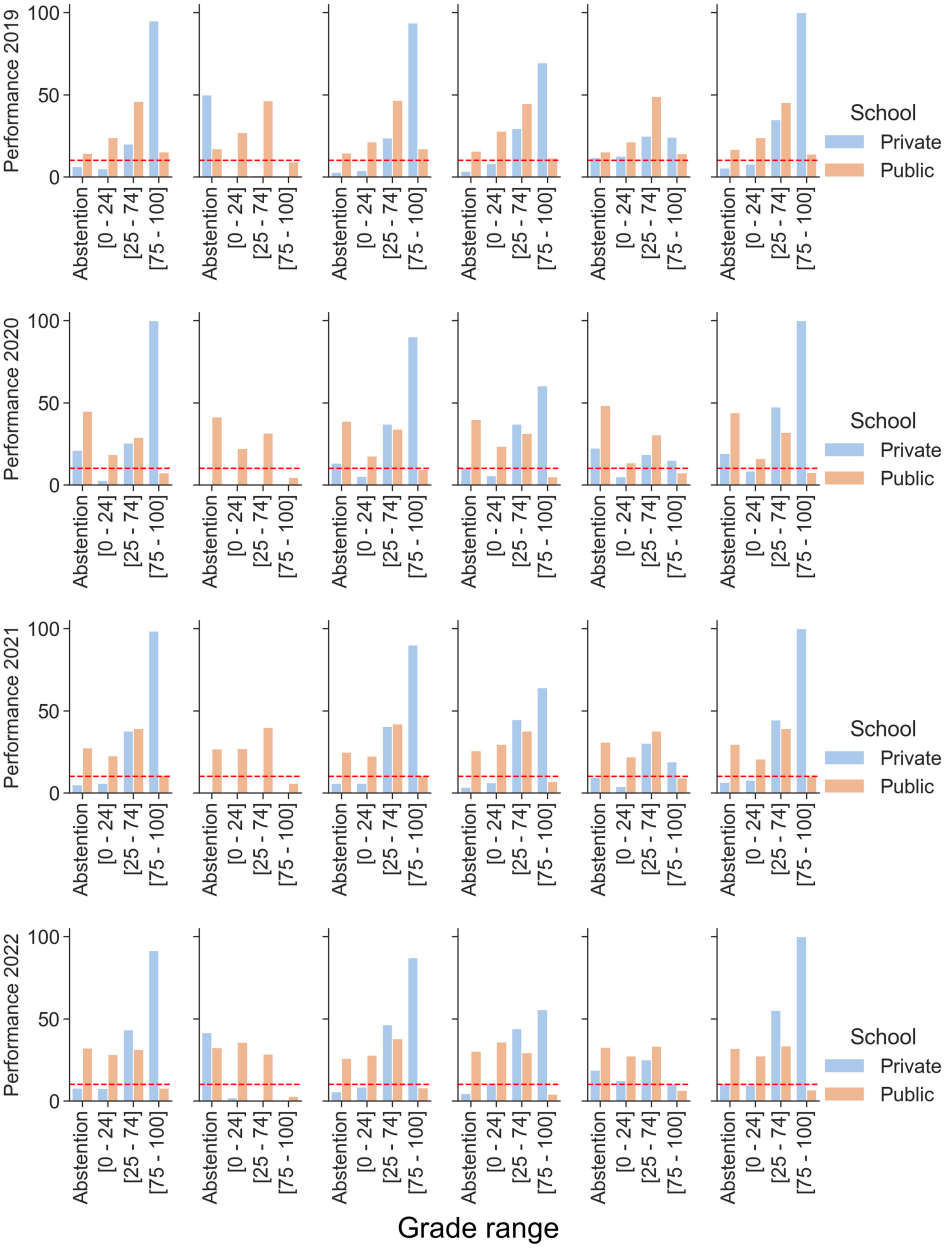
Comparison of student performance in Pará by Mesoregion in the ENEM (2019–2022).

Figure 3 suggests that regional differences played a crucial role in the impact of the pandemic on education. While some regions implemented strategies that helped mitigate dropout rates, others faced significant challenges, such as high dropout rates. Among the mesoregions of Pará presented in Figure 4, it stands out that only the Metropolitan Region of Belém and Northeast Pará significantly reduced ENEM dropout rates during the COVID-19 pandemic between 2020 and 2021. In contrast, the remaining regions maintained persistently high dropout rates, with percentages exceeding 20% during the same period.

The Marajó region was one of the most severely impacted after the onset of the COVID-19 pandemic. Notably, between 2020 and 2022, public school students exhibited a substantial decline in performance, with fewer than 10% achieving scores above 75% in assessments. Additionally, it is essential to highlight the significant increase in absenteeism among private school students during the ENEM. This trend may be related to mobility restrictions imposed by lockdowns and the closure of educational institutions on the island, as illustrated in Figure 4.

The findings suggest that the COVID-19 pandemic exacerbated existing socioeconomic inequalities, particularly concerning exam access and student performance. The forced transition to remote learning exposed structural weaknesses and highlighted the need for policies that ensure more equitable access to education, regardless of students' economic and regional conditions. Factors such as access to technological resources and the home environment proved to be decisive for academic success during this period.

The results of this study indicate that the COVID-19 pandemic significantly impacted students' participation and performance in the National High School Exam (ENEM), particularly in the more vulnerable regions of the state of Pará, Brazil. Students from low-income families with limited access to technological resources were the most affected, exhibiting the highest dropout rates between 2020 and 2021. These findings highlight the exacerbation of socioeconomic inequalities during the pandemic, with the interruption of in-person classes and the difficulty of adapting to remote learning primarily hindering public school students from lower-income backgrounds.

Access to technological resources, such as computers and the internet, played a crucial role in academic performance. Students from private schools, who often had better access to these resources, showed superior performance compared to their peers in public schools. The analysis also underscored the importance of parental education and occupation, which, when higher, contributed to better academic outcomes for students, suggesting the significance of a more structured family environment.

Additionally, the Bayesian network analysis and regional variations in Pará indicated that the pandemic affected the state's different mesoregions unevenly. While the Metropolitan Region of Belém and the Northeast of Pará were able to reduce dropout rates, other areas, such as the Island of Marajó, faced greater challenges, showing significantly reduced performance and higher abandonment rates.

### Limitations of the study

While the analysis provided valuable insights into the effects of the pandemic on academic performance, some limitations must be acknowledged. First, the use of Bayesian Networks, although effective in modeling probabilistic dependencies, relies on assumptions of conditional independence that may have been compromised in the emergency context of the pandemic. This could have led to distortions in the results, particularly when handling outlier data and variables influenced unpredictably by the pandemic. Additionally, the collection of data on socioeconomic and family factors may have been affected by incomplete information or access challenges during the period of restrictions. The analysis of regional variables also faces limitations, as the implementation of educational policies and local infrastructure in each mesoregion could have influenced the results unevenly.

In summary, while the findings provide a comprehensive view of the pandemic's impacts on the ENEM, future studies may need to address these limitations by expanding the analysis to include additional variables or more robust data collection methods, aiming to refine the models and provide a more detailed understanding of the factors influencing educational performance in times of crisis.

## Discussion

The results reveal the profound and unequal impact of the COVID-19 pandemic on academic performance and student participation in the ENEM in the state of Pará. A detailed analysis of the different mesoregions and the relationship between socioeconomic factors and performance highlights several trends and challenges that should be considered for the future of education in the region.

The data showed that the pandemic exacerbated existing inequalities, especially among low-income students and those attending public schools. The highest dropout rates were observed among participants with a family income of up to one minimum wage, highlighting the difficulties faced by families unable to adapt to remote learning due to a lack of technological resources and adequate infrastructure. This trend was particularly evident in Table 2, where low-income groups recorded the highest dropout rates during the peak pandemic (2020 and 2021). This scenario underscores the need for greater attention to inequality and health literacy issues, which are essential to support students' holistic development and education (de Oliveira et al., 2024).

This disparity reflects an urgent need for investments in digital infrastructure and educational support for low-income students. Public policies must ensure universal access to resources such as computers and the Internet to prevent economic inequalities from translating into disparities in educational opportunities.

As illustrated in Table 3, the analysis of academic performance revealed a strong correlation between access to technological resources and academic success during remote learning. Students with access to computers and the internet achieved significantly higher performance, with private school students registering a 20% increase in scores compared to their peers without these resources.

This finding highlights the importance of ensuring that all students, regardless of location or economic status, access tools that enable effective learning. Educational policies should prioritize the distribution of technological resources to minimize the impact of potential future school disruptions.

Regional analysis revealed significant disparities in the impact of the pandemic across the mesoregions of Pará. Figure 3 highlighted that while the Metropolitan Region of Belém and Northeast Pará managed to reduce dropout rates during the pandemic, other regions, such as Marajó and Southeast Pará, continued to face considerable challenges. These regions maintained high dropout rates, suggesting that factors such as local infrastructure, access to technology, and educational support were insufficient to ensure learning continuity.

According to Figure 4, fewer than 10% of public school students achieved scores above 75% between 2020 and 2022, while absenteeism in the ENEM significantly increased among private school students. This scenario may be explained by a combination of factors, including severe mobility restrictions imposed during lockdowns and the closure of educational institutions, which hindered students' access to exams and continuous learning.

This regional analysis demonstrates the need for a more specific, region-based approach to addressing educational inequalities. Support programs that consider each mesoregion's unique characteristics and challenges may be more effective than generic solutions, ensuring that more isolated and economically disadvantaged regions receive the necessary attention.

The results and discussions indicate the need for more inclusive and adaptive educational policies. The pandemic revealed that the educational system must be resilient and prepared to handle emergencies that may disrupt in-person learning. Investments in technology, teacher training for remote education, and programs for psychological and social support for students are essential to build a more robust and equitable educational system.

In summary, the analysis of ENEM data in Pará revealed not only the immediate impact of the COVID-19 pandemic on education but also systemic issues that need to be addressed moving forward. Economic inequalities, regional disparities, and limited resource access hinder educational equity. Public policies and private initiatives must work together to reduce these inequalities, ensuring that all students have equal opportunities for success, regardless of socioeconomic background or geographical location.

## Data Availability

Publicly available datasets were analyzed in this study. This data can be found at: https://www.gov.br/inep/pt-br/acesso-a-informacao/dados-abertos/microdados/enem.

## Footnotes

^1^ https://www.gov.br/inep/pt-br/acesso-a-informacao/dados-abertos/microdados/enem
